# Supplementary material for: Sphingosine-1-phosphate suppresses GLUT activity through PP2A and counteracts hyperglycemia in diabetic red blood cells
Source: Nat Commun. 2023 Dec 14;14:8329. doi: 10.1038/s41467-023-44109-x (PMC10721873; doi:10.1038/s41467-023-44109-x)
Supplement: Supplementary file 1 — Supplementary Information [file 41467_2023_44109_MOESM1_ESM.pdf]

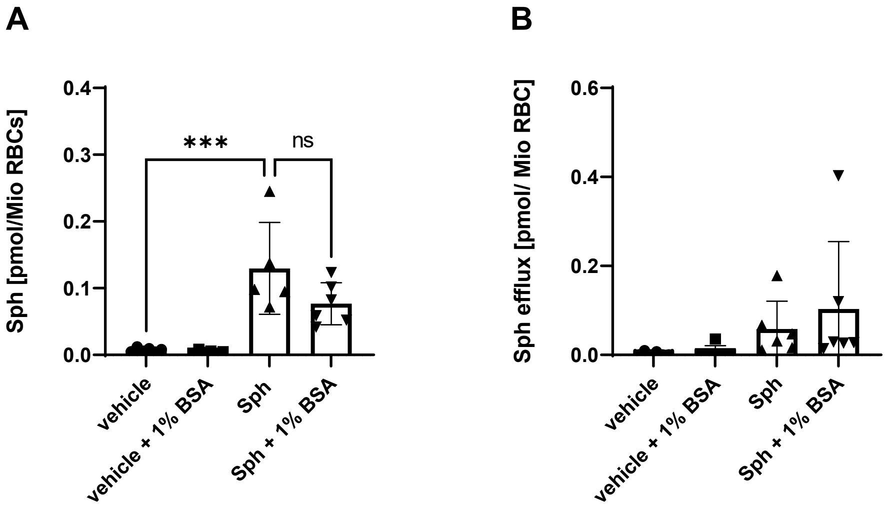

Supplementary figure 1: Sphingosine does not accumulate in RBC or efflux to BSA.
A) Sphingosine levels in RBC and **B)** in supernatants with or without 1% BSA after 30 min following a 30 min incubation with 1 µM sph (n=6). Data are presented as mean±sd and tested with paired one-way ANOVA; ns= not significant; p***<0.001

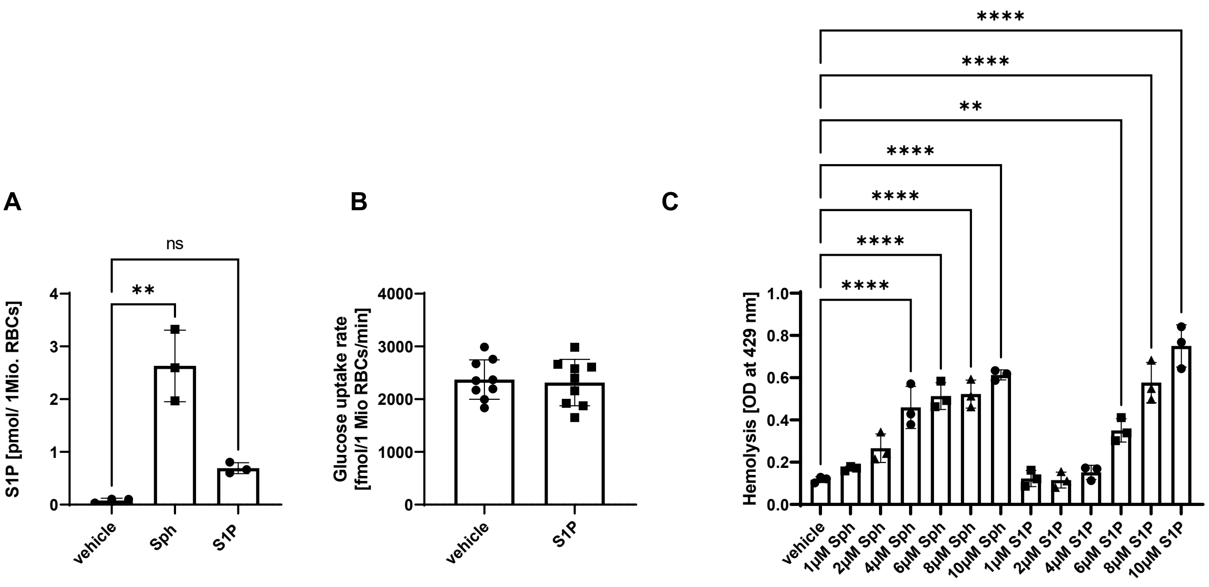

Supplementary figure 2: Effect of extracellular S1P on RBC S1P content, glucose uptake hemolysis. **A)** Iintracellular S1P levels in RBC after incubation with 1µM, Sph, S1P and vehicle (MeOH, 0,1 %) for 30 min at 37°C (n=3 each). **B)** Glucose uptake rate in RBC after incubation with 1 µM S1P and vehicle (MeOH, 0,1 %) for 30 min at 37°C (n=8 each). **C)** Hemolysis in murine RBC supernatants after incubation with Sph and S1P in 1, 2, 4, 6 and 8 µM (30 min for 37°C) as measured by optical density of haemoglobin at the wavelength of 429 nm (n=3 each). Data are presented as mean±sd and tested with two-tailed paired t-test (A) and one-way ANOVA (B + C); ns= not significant; p**<0.01; p****<0.0001.

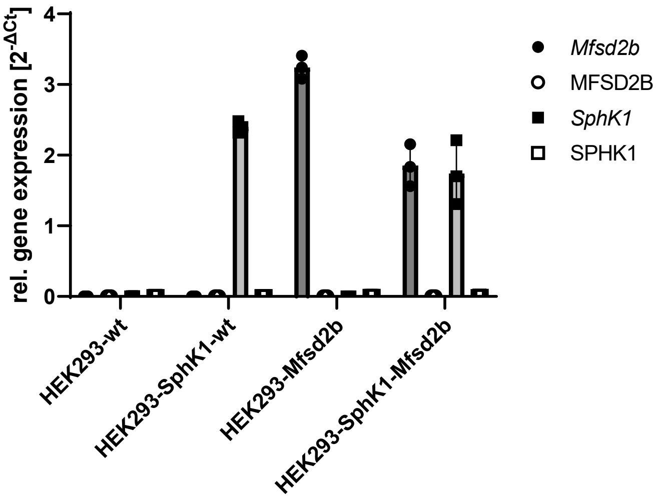

Supplementary figure 3: SphK1 and Mfsd2b overexpression in Hek293 cells. Relative gene expression of murine Sphk1 and Mfsd2b in HEK293 cells stably overexpressing Sphk1, Mfsd2b or both by RT/real-time PCR. Human SPHK1 and MFSD2B expression was also quantified to assess basal levels in the Hek293 cell line and all transfectants. Data are normalised to GAPDH and shown as $2^{-\Delta Ct}$ (n=3).

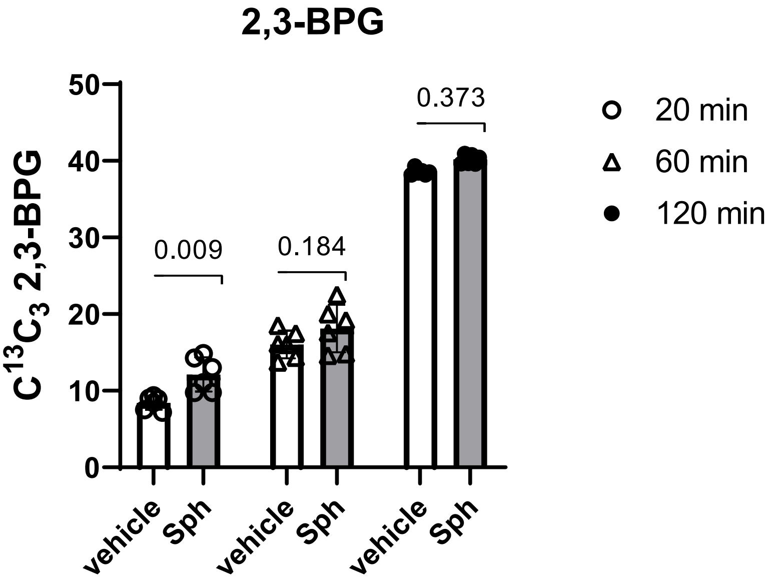

Supplementary figure 4: 2,3-BPG relative relative exchange (time kinetic) in RBC incubated with 1,2,3-$^{13}C_3$ glucose with and without sphingosine preincubation. Data are presented as mean±sd and tested with two-way ANOVA.

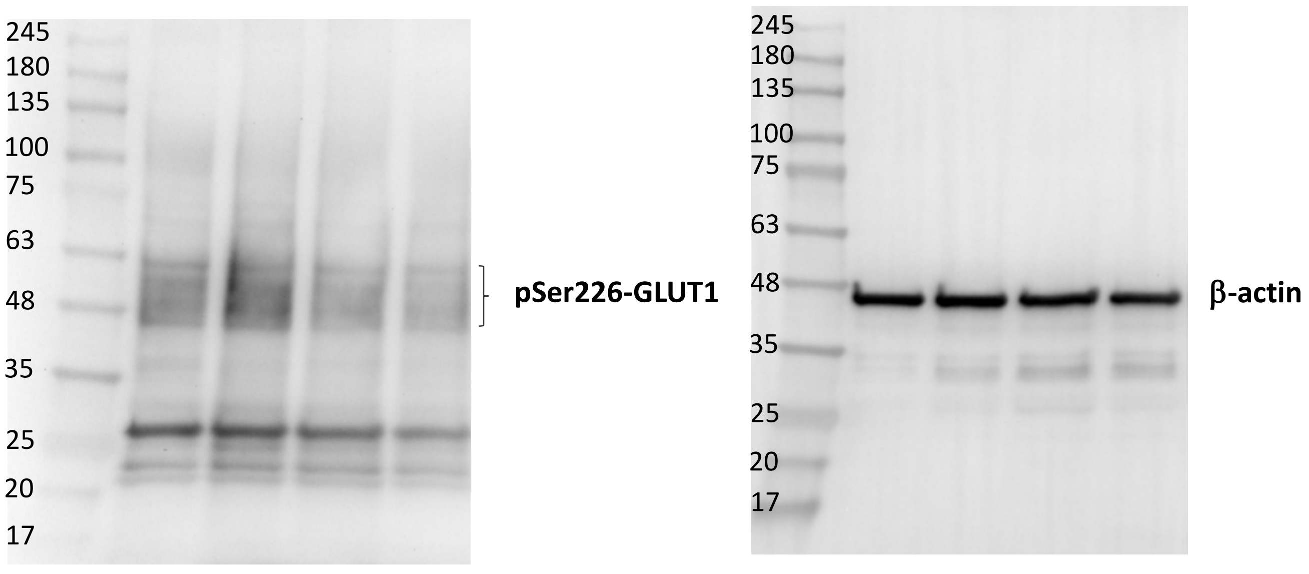

Supplementary figure 5: Western blotting for GLUT1 Serine 226 phosphorylation and beta-tubulin (uncropped blots). Excerpts are shown in Fig. 6G. Molecular weight according to CozyHi™ Prestained Protein Ladder (highQu GmbH). Membranes were probed for phospho-Serine 226-GLUT1 (ABN991; 1:200) and beta-actin (A1978; 1:100), both from Sigma-Aldrich, St. Louis, USA. These are the uncropped Western blots of Figure 6.

Supplementary table 1 : Patient characteristics. Data are presented as mean±sd and tested with two-tailed unpaid t-test.

	control	T2DM	p value
HbA1c	5.24	8.95	0.000001
Male sex	8	10	>0.05
Female sex	5	3	>0.05
age	62 (±18.4)	74 (±10.0)	>0.05
Weight [kg]	79 (± 13.9)	83 (± 16.3)	>0.05
Body mass index	27 (± 4.6)	28 (± 5.9)	>0.05
