# Supplementary material for: Sphingosine-1-phosphate suppresses GLUT activity through PP2A and counteracts hyperglycemia in diabetic red blood cells
Source: Nat Commun. 2023 Dec 14;14:8329. doi: 10.1038/s41467-023-44109-x (PMC10721873; doi:10.1038/s41467-023-44109-x)
Supplement: Supplementary file 3 — Reporting Summary [file 41467_2023_44109_MOESM3_ESM.pdf]

nature portfolio

Corresponding author(s): Bodo Levkau

Last updated by author(s): Nov 10, 2023

Reporting Summary

Nature Portfolio wishes to improve the reproducibility of the work that we publish. This form provides structure for consistency and transparency in reporting. For further information on Nature Portfolio policies, see our Editorial Policies and the Editorial Policy Checklist.

Statistics

For all statistical analyses, confirm that the following items are present in the figure legend, table legend, main text, or Methods section.

n/a	Confirmed	
☐	☒	The exact sample size (*n*) for each experimental group/condition, given as a discrete number and unit of measurement
☐	☒	A statement on whether measurements were taken from distinct samples or whether the same sample was measured repeatedly
☐	☒	The statistical test(s) used AND whether they are one- or two-sided<br>*Only common tests should be described solely by name; describe more complex techniques in the Methods section.*
☒	☐	A description of all covariates tested
☐	☒	A description of any assumptions or corrections, such as tests of normality and adjustment for multiple comparisons
☐	☒	A full description of the statistical parameters including central tendency (e.g. means) or other basic estimates (e.g. regression coefficient) AND variation (e.g. standard deviation) or associated estimates of uncertainty (e.g. confidence intervals)
☐	☒	For null hypothesis testing, the test statistic (e.g. *F*, *t*, *r*) with confidence intervals, effect sizes, degrees of freedom and *P* value noted<br>*Give P values as exact values whenever suitable.*
☒	☐	For Bayesian analysis, information on the choice of priors and Markov chain Monte Carlo settings
☒	☐	For hierarchical and complex designs, identification of the appropriate level for tests and full reporting of outcomes
☐	☒	Estimates of effect sizes (e.g. Cohen's *d*, Pearson's *r*), indicating how they were calculated

Our web collection on statistics for biologists contains articles on many of the points above.

Software and code

Policy information about availability of computer code

Data collection	Plate reader MARS Software, BMG LABTECH GmbH, Offenburg, Germany<br>LC-MS/MS metabolome primary data: LabSolutions 5.99, Shimadzu Deutschland GmbH, Duisburg, Germany<br>Flow cytometry data: Gallios Cytometer 1.2 Data Acquisition & Analysis Software
Data analysis	GraphPad PRISM Software 9.3.1, Gallios Cytometer 1.2 Data Analysis Software, BMG plate reader MARS Analysis Software, Shimadzu LabSolutions 5.99

For manuscripts utilizing custom algorithms or software that are central to the research but not yet described in published literature, software must be made available to editors and reviewers. We strongly encourage code deposition in a community repository (e.g. GitHub). See the Nature Portfolio guidelines for submitting code & software for further information.

Data

Policy information about availability of data

All manuscripts must include a data availability statement. This statement should provide the following information, where applicable:

- Accession codes, unique identifiers, or web links for publicly available datasets
- A description of any restrictions on data availability
- For clinical datasets or third party data, please ensure that the statement adheres to our policy

The data supporting this study can be found in the figures and supplementary information. Source data are provided with this paper.

Research involving human participants, their data, or biological material

Policy information about studies with human participants or human data. See also policy information about sex, gender (identity/presentation), and sexual orientation and race, ethnicity and racism.

Reporting on sex and gender	Information on sex is provided in supplemental table 1. Findings apply to both sexes. Sex was not considered in study design because of similar S1P values in males and females. The mechanism of this study is independent of the sex of the mice. In humans, sex determined as assigned. Information has not been collected on disaggregated sex and gender data. Sex-based analysis was not performed due to low numbers (male sex n=8 in the control group and b=10 in the 2TD group; female sex n=5 in the control group and n=3 in the 2TD group).
Reporting on race, ethnicity, or other socially relevant groupings	Race and ethnicity or other social relevant groupings were not part of the inclusion criteria.
Population characteristics	A detailed description of covariate-relevant patient characteristics can be found in supplemental table 1: sex, weight, age, BMI and diabetes type II
Recruitment	Strudy design: Prospective, Monocentric, Observational; All-comers design<br>Inclusion criteria age ≥ 18 years and written informed consent. Exclusion criteria: Active cancer with life expectancy < 12 months; Coagulopathies. Sex of participants as on admission forms (assigned). No gender reporting.
Ethics oversight	The study was in accordance with the Declaration of Helsinki and was approved by the University of Düsseldorf Ethics Committee. All participants have given written informed consent without monetary compensation

Note that full information on the approval of the study protocol must also be provided in the manuscript.

Field-specific reporting

Please select the one below that is the best fit for your research. If you are not sure, read the appropriate sections before making your selection.

☒ Life sciences ☐ Behavioural & social sciences ☐ Ecological, evolutionary & environmental sciences

For a reference copy of the document with all sections, see nature.com/documents/nr-reporting-summary-flat.pdf

Life sciences study design

All studies must disclose on these points even when the disclosure is negative.

Sample size	As this was an explorative study, sample size was chosen due to previous experience in differences in S1P levels in RBCs observed in murine models where for acceptable SD we needed in general 5-6 n.
Data exclusions	In Fig 2 a/2b one data point was excluded as "outlier". The excluded data point is marked with an * in the raw date file.
Replication	Each data point corresponds to the measured value associated with an individual or a cell experiment. All data could be replicated independently by more than one investigator. The total n are always indicated.
Randomization	For the murine experiments inbred mouse strains were used and mice assigned randomly. For the human data randomization was not applicable since patients were grouped according to their HbA1c levels (normal/abnormal)
Blinding	The investigators were not blinded to treatment and analysis. More than one investigator have performed experiments and data pooled irrespective of investigator. An investigatior blinded on group identity has reproduced and confirmed statistical differences between groups.

Reporting for specific materials, systems and methods

We require information from authors about some types of materials, experimental systems and methods used in many studies. Here, indicate whether each material, system or method listed is relevant to your study. If you are not sure if a list item applies to your research, read the appropriate section before selecting a response.

Materials & experimental systems

n/a	Involved in the study
☐	☒ Antibodies
☐	☒ Eukaryotic cell lines
☒	☐ Palaeontology and archaeology
☐	☒ Animals and other organisms
☒	☐ Clinical data
☒	☐ Dual use research of concern
☒	☐ Plants

Methods

n/a	Involved in the study
☒	☐ ChIP-seq
☐	☒ Flow cytometry
☒	☐ MRI-based neuroimaging

Antibodies

Antibodies used	Sphingomab, courtesy of R. Sabbadini. Phospho-Serine 226-GLUT1 (Thermo Fisher Scientific, Waltham, USA) and beta-actin (Sigma-Aldrich, St. Louis, USA).
Validation	Sphingomab, courtesy of R. Sabbadini. First described in O'Brien N, Jones ST, Williams DG, Cunningham HB, Moreno K, Visentin B, et al. Production and characterization of monoclonal anti-sphingosine-1-phosphate antibodies. J Lipid Res. 2009;50:2245–2257. Phospho-Serine 226-GLUT1 (ABN991; 1:200) and beta-actin (A1978; 1:100), both from Sigma-Aldrich, St. Louis, USA. Validation at manufacturer's homepage product sheets.

Eukaryotic cell lines

Policy information about cell lines and Sex and Gender in Research

Cell line source(s)	HEK293 cells overexpressing mouse Mfsd2b in vector ORF-MFSD2B (Origene) were provided by Prof. Dr. Markus Gräler, University Hospital Jena. HEK293 Mfsd2b and HEK293 control cells were stably transfected with murine Sk1 (Origene) cloned into pCDNA4-zeo mammalian expression vector and transfected using FuGENE (Promega) by our labatory.
Authentication	Expression of Mfsd2b and Sk1 were confirmed by qRT-PCR. No others were used.
Mycoplasma contamination	Cell lines were not testet for mycoplasma contamination
Commonly misidentified lines (See ICLAC register)	none

Animals and other research organisms

Policy information about studies involving animals; ARRIVE guidelines recommended for reporting animal research, and Sex and Gender in Research

Laboratory animals	C57BL6/J, both sexes, 12-18 weeks.<br>C57BL6/J with 4-deoxypyridoxine (DOP) 3mg/l (0.5mg per kg body weight per day) for three weeks, both sexes, 12-18 weeks when starting the DOP treatment<br>C57BL6/J with high caolory diet (60% energy from fat), male, 16-18 weeks when<br>SphK1-/- mice, both sexes, 12-18 weeks when starting the treatment<br>Sgpl vav-cre mice (generated by crossing Sgpl flox/flox mice (A. Billich, Novartis) with Commnd10Tg(vav1-iCre)A2Kio/J vav Cre transgenic mice), both sex, 12-18 weeks<br>Mfsd2b-/- , both sexes, 12-18 weeks.<br>All mice were kept at 12-hour light/dark cycle, ambient temperature 20-24°C, 45-65% humidity, water provided ad libitum, eutanasia by isoflurane.
Wild animals	This study did not involve field-collected samples.
Reporting on sex	Sex was not considered in the study design. Mice of both sexes were used. Sex-based analysis was not performed due to low numbers.
Field-collected samples	n/a
Ethics oversight	All mice experiments were approved by Landesamt für Natur, Umwelt und Verbraucherschutz Nordrhein-Westfalen (LANUV NRW) and in accordance with the European Convention for the Protection of Vertebrate Animals used for Experimental and other Scientific Purposes (Council of Europe Treaty Series No. 123) and 2010/63/EU (Az 84-02.04.2017.A097, 84-02.04.2017.A087 and 81-02.04.2021.A048).

Note that full information on the approval of the study protocol must also be provided in the manuscript.

Plants

Seed stocks

Report on the source of all seed stocks or other plant material used. If applicable, state the seed stock centre and catalogue number. If plant specimens were collected from the field, describe the collection location, date and sampling procedures.

Novel plant genotypes

Describe the methods by which all novel plant genotypes were produced. This includes those generated by transgenic approaches, gene editing, chemical/radiation-based mutagenesis and hybridization. For transgenic lines, describe the transformation method, the number of independent lines analyzed and the generation upon which experiments were performed. For gene-edited lines, describe the editor used, the endogenous sequence targeted for editing, the targeting guide RNA sequence (if applicable) and how the editor was applied.

Authentication

Describe any authentication procedures for each seed stock used or novel genotype generated. Describe any experiments used to assess the effect of a mutation and, where applicable, how potential secondary effects (e.g. second site T-DNA insertions, mosiacism, off-target gene editing) were examined.

Flow Cytometry

Plots

Confirm that:

☐ The axis labels state the marker and fluorochrome used (e.g. CD4-FITC).

☒ The axis scales are clearly visible. Include numbers along axes only for bottom left plot of group (a 'group' is an analysis of identical markers).

☐ All plots are contour plots with outliers or pseudocolor plots.

☒ A numerical value for number of cells or percentage (with statistics) is provided.

Methodology

Sample preparation	Surface Glut1 expression in human RBC was measured using the receptor-binding domain of a recombinant envelope glycoprotein from human T lymphotrophic virus (HTLV) fused to EGFP (H2 EGFP). RBCs were incubated with H2-EGFP in PBS containing 0.33 mg/ml BSA and 1 mM EDTA at 37°C for 20 min. Afterwards, RBCs are washed twice with PBS and resuspended in 400 µl FACS buffer (NaCL 140 mM, H2Na2O6P 13 mM, EDTA 1.075 mM, KCL 5.5 mM, NaH2PO4·H2O 1.6 mM, NaF 75.55 mM). Cell surface GLUT4 flow cytometry was performed with an antibody that recognizes an extracellular domain of the transporter (LM048) as published. On site validation was performed with the L6-GLUT4myc rat myoblast cell line (Kerafast, Boston, USA) that overexpresses GLUT4.
Instrument	Gallios Flow Cytometer, Beckman Coulter, Brea, USA
Software	Measurment and Analysis of flow cytometry data: Callios Cytometer 1.2 Data Acquisition & Analysis Software, Beckman Coulter, Brea, USA.<br>Illustration of the overlay is made by Kaluza Anakysis Flow Cytometry Software 2.1.2, Beckman Coulter, Brea, USA.
Cell population abundance	Cells of interest were selected via FSC/SSC (size) and gating of GFP positive cells.
Gating strategy	Gating for total cell population by FSC/SSC (size). Gating for GPF positive cells. A simple histogram overlay is provided in the figures. No need for gating strategy. There is none.

☐ Tick this box to confirm that a figure exemplifying the gating strategy is provided in the Supplementary Information.
